# Large Language Models in Medical and Dental Education: A Cross-Sectional Comparison of AI-Generated and Faculty-Authored Prosthodontic Materials

**DOI:** 10.3390/dj14050249

**Published:** 2026-04-23

**Authors:** Alexia-Ecaterina Cârstea, Lucian-Toma Ciocan, Vlad-Gabriel Vasilescu, Ana-Maria Cristina Țâncu, Marina Imre, Andreea-Cristiana Didilescu, Silviu-Mirel Pițuru

**Affiliations:** 1 Department of Dental Prostheses Technology, Faculty of Dentistry, “Carol Davila” University of Medicine and Pharmacy, Dionisie Lupu Street, No. 37, District 2, 020021 Bucharest, Romania; alexia-ecaterina.carstea2023@stud.umfcd.ro; 2 Department of Prosthodontics, Faculty of Dentistry, “Carol Davila” University of Medicine and Pharmacy, 37 Dionisie Lupu Street, District 2, 020021 Bucharest, Romania; anamaria.tancu@umfcd.ro (A.-M.C.Ț.); marina.imre@umfcd.ro (M.I.); 3 Department of Embriology and Microbiology, Faculty of Dentistry, “Carol Davila” University of Medicine and Pharmacy, 37 Dionisie Lupu Street, District 2, 020021 Bucharest, Romania; andreea.didilescu@umfcd.ro; 4 Department of Organization, Professional Legislation and Management of the Dental Office, Faculty of Dental Medicine, “Carol Davila” University of Medicine and Pharmacy, 17-23 Plevnei Street, 020021 Bucharest, Romania; silviu.pituru@umfcd.ro

**Keywords:** Large Language Models, artificial intelligence, prosthodontics

## Abstract

**Background/Objectives**: This study aimed to compare AI-generated educational material with faculty-authored content in Dental Prostheses Technology, evaluating perceived clarity, accuracy, structure, usefulness, and overall instructional quality across different age and professional groups. **Methods**: An analytical cross-sectional study was conducted using two versions of the first three chapters of a prosthodontics textbook: the original faculty-authored text and a reformulated version generated by ChatGPT 5.2 (OpenAI). Images were removed and formatting standardized to ensure a text-only comparison. An anonymized online questionnaire based on a five-point Likert scale assessed clarity, accuracy, readability, usefulness and structure. To reduce potential bias, participants were unaware of the authorship of the evaluated materials (human-authored vs. AI-generated). A total of 130 participants independently reviewed both documents. Data were analyzed using Wilcoxon signed-rank, Mann–Whitney U, and Friedman tests. **Results**: Both materials received favorable evaluations across all dimensions. The AI-generated version demonstrated a statistically significant advantage in clarity (Z = −2.107, *p* = 0.035; r = 0.19), while no significant differences were observed for structure, accuracy, readability, or usefulness. Generational differences emerged: younger participants valued improved clarity but reported reduced usefulness, mid-career participants showed the greatest improvement in perceived accuracy, and senior professionals reported substantial gains in usefulness and readability. **Conclusions**: AI-generated educational material demonstrates pedagogical equivalence to faculty-authored content, with clarity representing its principal advantage. Large language models may serve as effective complementary tools in dental education, particularly for restructuring complex content.

## 1. Introduction

Dental prostheses technology constitutes a foundational discipline within dental education, distinguished by its dual demand for mastery of complex theoretical frameworks and advanced practical competencies. The effective delivery of educational content in this field presents persistent challenges for academic faculty, particularly to maintaining conceptual depth, terminological precision, and pedagogical structure across diverse student cohorts. These challenges are further compounded by the resource-intensive nature of developing high-quality instructional materials that simultaneously address content accuracy and learner engagement. It is within this educational context that the emergence of artificial intelligence-based tools warrants serious examination as a potential supplementary resource in specialized, high-stakes academic training.

The integration of Large Language Models (LLMs), including ChatGPT, into higher education has become an area of increasing academic inquiry, particularly within disciplines that require the simultaneous mastery of complex theoretical frameworks and advanced practical competencies, such as medicine and dentistry [1,2,3,4,5,6,7,8]. The rapid advancement of artificial intelligence technologies has prompted critical examination of their potential role in supporting educational processes, curriculum development, and knowledge dissemination in academically rigorous environments [9,10,11,12].

Large Language Models represent a class of deep learning systems built upon transformer-based neural architectures, trained on vast corpora of textual data and capable of performing a wide spectrum of natural language processing tasks, including content generation, summarization, question answering, and contextual reasoning [13,14]. Since the introduction of foundational models such as BERT and the GPT series, there has been rapid evolution in both the scale and application of LLMs across multiple domains [15]. The release of ChatGPT in November 2022 marked a pivotal moment in the public accessibility of generative AI, significantly transforming the landscape of educational technology and prompting widespread investigation into its empirical applications, benefits, and limitations [15]. In educational contexts, LLMs have demonstrated capacity across several instructional functions: early integrations of LLMs into educational settings have shown promising results in automatically generating educational content, providing contextual feedback, and supporting student knowledge acquisition across disciplines including medicine [16]. The potential of LLMs to serve as intelligent tutoring systems, capable of delivering individualized support, engaging students in reflective dialogue, and facilitating active recall, has been recognized as a significant opportunity for higher education, particularly at the postgraduate and professional training levels [17,18].

Studies have evaluated ChatGPT’s performance in generating answers related to removable dental prostheses and tooth-supported fixed dental prostheses, finding limited reliability in prosthesis fabrication-related responses, while LLMs have shown more consistent accuracy in periodontal classification tasks [19]. In prosthodontics specifically, multiple investigations have benchmarked LLM performance against standardized professional examinations. ChatGPT has shown promising potential as an educational tool for prosthodontics residents by effectively addressing board-style questions; however, a significant presence of inaccuracies in its current prosthodontics knowledge base warrants caution and the supplementation of AI-generated content with evidence-based sources [20]. While LLM performance has shown promise in general dental examinations, their effectiveness in subspecialties such as prosthodontics remains underexplored, and assessing AI accuracy in this focused domain may reveal important limitations and potential as a supplementary tool for postgraduate preparation [21].

This study investigates the application of LLMs in the generation of educational content for dental prostheses technology [22,23,24,25,26]. The research undertakes a comparison between teaching materials generated by an AI-based language model and those authored by academic faculty members, with the objective of evaluating their relative performance across multiple pedagogical and content-related dimensions [27].

By providing an evidence-based evaluation of AI-assisted content generation, this research aims to contribute to the broader discourse on the responsible integration of artificial intelligence in medical and dental education [28,29,30,31,32]. The findings are intended to inform educators, curriculum designers, and policy-makers regarding the potential benefits, limitations, and implications of employing LLMs as supplementary tools in the delivery of specialized, high-stakes educational content [33,34,35,36].

## 2. Materials and Methods

The original course material for the subject Dental Prostheses Technology was revised and expanded using a Large Language Model (LLM), specifically ChatGPT 5.2 OpenAI (San Francisco, CA, USA), with the objective of creating an AI-generated version suitable for comparison with faculty-authored educational content. An anonymized questionnaire was designed to evaluate participants’ perceptions regarding the clarity, accuracy, accessibility, and educational usefulness of the AI-generated material. The survey was distributed to a total of 130 participants, divided into four groups: medical students, dental students, dental faculty members, and individuals from non-medical fields (such as engineering, business, the military, and retirees). Each participant was asked to review the content and complete the questionnaire independently. Participants were not informed which version of the text was faculty-authored and which was generated by artificial intelligence, in order to minimize bias and ensure objective evaluation. All responses were collected anonymously to preserve the integrity and impartiality of the data.

### 2.1. Study Design

This analytical, cross-sectional study was designed to compare faculty-authored educational content in dental prostheses technology with an adapted version generated by a Large Language Model (LLM).

### 2.2. Source Materials

#### 2.2.1. Faculty-Authored Text

The original material consisted of the first three chapters of the textbook “Fixed Dental Prostheses Technology” [37], published in 2024. The textbook serves as the primary learning resource for second-year dental students of the Faculty of Dentistry at the “Carol Davila” University of Medicine and Pharmacy and has been used by four cohorts since its publication. The content is written in a formal, technically precise style and presents theoretical principles and laboratory procedures in a traditional narrative format, with minimal use of tables.

#### 2.2.2. LLM-Generated Text

The comparative material was produced using ChatGPT 5.2 OpenAI (San Francisco, CA, USA), which reformulated the same three chapters of the original textbook and reorganized the content to enhance clarity. In contrast to the original text, the LLM-generated version introduced tables and structured elements that were not present in the source material.

#### 2.2.3. AI Text Generation Procedure

The LLM was instructed to reformulate the first three chapters of the original textbook while preserving all essential scientific information. Prompts emphasized maintaining an academic tone, improving conceptual clarity, and restructuring the content to enhance readability and retention for students. No external corrections or additions were introduced, ensuring that the resulting text reflected the model’s autonomous output under these defined constraints.

To ensure a strictly textual comparison, all images and visual elements were removed from the original textbook prior to processing. The same restriction was applied to the LLM-generated text, which was produced without images, diagrams, or graphical aids. Formatting was standardized across both versions to minimize potential bias attributable to visual presentation, allowing the evaluation to focus on the written content itself.

### 2.3. Participants

#### 2.3.1. Eligibility Criteria and Ethical Approval

Participants were eligible for inclusion in the study if they met the following criteria: they were adults aged 18 years or older, had sufficient proficiency in English to understand and complete it accurately, and had prior exposure to or experience with the analyzed content. Only fully completed questionnaires were considered valid for analysis.

This study was approved by the Research Ethics Subcommittee of the “Carol Davila” University of Medicine and Pharmacy, Bucharest, Romania (Registration number: 3963; date: 20 February 2026). The research was conducted in accordance with the Declaration of Helsinki.

#### 2.3.2. Group Composition and Rationale

A total of 130 participants were enrolled, distributed across four groups. Dental students represented the primary intended audience of the evaluated material, as the source textbook is used in the second year of the dental curriculum; their inclusion was therefore central to the study’s objective. Medical students were included on the basis that dental education in Romania is institutionally integrated within medical universities, and medical students share substantial foundational biomedical training with dental students, making their perceptions of scientific accuracy and conceptual clarity relevant for comparative purposes. Dental faculty members were included as subject-matter experts capable of providing specialist-level evaluation of content accuracy and pedagogical appropriateness. Participants from non-medical fields (including engineering, business, and other professions) were included specifically to assess dimensions of readability and general accessibility—that is, the degree to which the materials are comprehensible to an educated lay audience—rather than to evaluate disciplinary accuracy or technical terminology. It is acknowledged that this group is not positioned to assess clinical accuracy or domain-specific terminology; accordingly, their evaluative contribution is interpreted as pertaining exclusively to readability and structural organization. This distinction is reflected in the stratified analyses presented in Section 3.

Participants were categorized into three predefined age groups: 18–28 years, 29–44 years, and 45 years and above [38,39,40]. These age intervals were selected to allow for comparative analysis across different generational cohorts. Participants aged 18–28 years predominantly corresponded to Generation Z [41], comprising individuals in the early stages of higher education or initial professional training, typically characterized by high exposure to digital technologies and contemporary learning environments [42,43]. The 29–44 years age group aligned with Millennials (Generation Y), representing individuals in mid-career academic or clinical stages, including postgraduate training, residency programs, and early academic appointments [43]. The 45 years and above category constituted a heterogeneous group encompassing both Generation X and Baby Boomer cohorts [43]. This group included senior academics and experienced professionals with extensive clinical and teaching backgrounds.

This age-based and generational classification framework enabled a structured examination of potential differences in perceptions, engagement, and evaluative criteria across distinct age and career stages, while also acknowledging the methodological limitations inherent in age-group approximations (Table 1). The limitations of this classification, including its reliance on age ranges rather than precise birth-year data, are acknowledged in the Limitations section.

#### 2.3.3. Recruitment Procedure

Participants were recruited using a convenience sampling strategy between October 2025 and January 2026. Dental and medical students were approached directly by members of the research team through institutional academic platforms and course communication channels affiliated with the Faculty of Dentistry and the Faculty of Medicine at “Carol Davila” University. Participants from non-medical fields were recruited through professional networks and groups. All potential participants received a standardized information message describing the general purpose of the study (evaluation of educational content), the voluntary nature of participation, the anonymous handling of responses, and an estimated time requirement for completion. No financial or academic incentives were offered.

It is acknowledged that convenience sampling introduces potential selection bias and limits the generalizability of findings to broader populations. Furthermore, the unequal group sizes, particularly the smaller number of faculty participants relative to student groups, reflect the practical constraints of recruiting specialized academic populations and should be considered when interpreting the results of between-group comparisons.

### 2.4. Data Collection and Survey Instrument

Data were collected through an online questionnaire administered alongside two attached documents: Text 1 (the faculty-authored course material) and Text 2 (the LLM-generated version). Participants were explicitly asked to read each document carefully and to complete the questionnaire only after reviewing both materials. Completion was individual, without time constraints, and without external assistance. Participants were not informed which text was faculty-authored and which was AI-generated, in order to minimize evaluative bias [44].

The questionnaire assessed six dimensions: clarity (ease of understanding of the written content); accuracy (perceived correctness and reliability of the information presented); readability—referred to as accessibility in the original instrument—(the degree to which the text is approachable and comprehensible to the intended reader, independent of prior domain knowledge); usefulness (perceived relevance and practical value for learning); structure (logical organization and coherence of the content). The questionnaire was distributed online between October 2025 and January 2026. It was administered using digital survey platforms and shared electronically professional communication channels.

Responses were recorded on a five-point Likert scale: 1 = Strongly Disagree, 2 = Disagree, 3 = Neutral, 4 = Agree, 5 = Strongly Agree. This scale was chosen to capture the intensity of respondents’ attitudes and perceptions in a standardized and quantifiable manner, facilitating statistical analysis and comparison across the evaluated dimensions.

### 2.5. Statistical Analysis

Descriptive statistics (means, standard deviations, frequencies) were computed for all questionnaire dimensions. Between-group comparisons were conducted using appropriate non-parametric tests, given the ordinal nature of Likert-scale data and the unequal group sizes. Correlation analyses were performed using Spearman’s rank-order correlation coefficient to examine associations between evaluative dimensions. Internal consistency of the questionnaire was assessed via Cronbach’s Alpha. The adequacy of the sample size for the planned correlation analyses was evaluated a priori using G*Power (version 3.1), targeting a medium effect size (r = 0.30) at a significance level of α = 0.05 with 80% power, which indicated a minimum required sample of 84 participants; the enrolled sample of 130 participants was therefore considered sufficient. Statistical analyses were performed using IBM SPSS Statistics (version 29.0.2.0, IBM Corp., Armonk, NY, USA). Advanced graphical visualization was conducted using OriginPro (version 2024, OriginLab Corporation, Northampton, MA, USA), and initial data screening and dataset preparation were performed in Microsoft Excel.

## 3. Results

### 3.1. Participant Characteristics

A total of 130 participants completed the questionnaire. The sample comprised four professional groups: dental students (*n* = 45, 34.6%), medical students (*n* = 44, 33.8%), participants from non-medical fields (*n* = 27, 20.8%), and dental faculty members (*n* = 14, 10.8%). With respect to age distribution, 94 participants (72.3%) were aged 18–28 years, 18 (13.8%) were aged 29–44 years, and 18 (13.8%) were aged 45 years or above. The distribution of participants across professional categories and age groups is presented in Table 1.

### 3.2. Descriptive Statistics

Throughout the analyses, Text 1 refers to the faculty-authored course material and Text 2 refers to the version of the same content generated by the Large Language Model. Participants evaluated both texts positively across all dimensions. For Text 1, mean scores ranged from 3.75 (readability) to 4.12 (clarity), indicating moderate to high appreciation across all evaluated criteria. Text 2 received similarly favorable evaluations, with mean values between 3.76 (usefulness) and 4.28 (clarity).

A direct comparison of mean scores indicates that Text 2 was generally rated slightly higher than Text 1 on most dimensions, particularly accuracy (4.16 vs. 4.05) and clarity (4.28 vs. 4.12). Readability and usefulness were comparable between the two texts, with marginally higher values for Text 2. Standard deviations across items ranged between 0.76 and 1.16, indicating moderate response variability.

A summary of mean scores and standard deviations for both texts across all evaluated dimensions is presented in Table 2.

The Cronbach’s Alpha coefficients indicated good internal consistency for both evaluated texts. Specifically, Text 1 demonstrated a Cronbach’s alpha of 0.841, while Text 2 showed a slightly higher value of 0.856. These results suggest that the set of items used for evaluation was reliable for both the faculty-authored and LLM-generated materials.

### 3.3. Age Group Differences

Although the primary intended audience of the evaluated material is dental students, the broader participant sample encompassed a wide age and professional range. Age-group analyses were therefore conducted to examine whether evaluative patterns differed systematically across generational cohorts, given documented differences in attitudes toward AI-generated content across age groups. These findings should be interpreted in the context of the unequal group sizes noted in Table 1.

#### 3.3.1. Age Group 18–28 Years (Generation Z)

**Figure 1:**
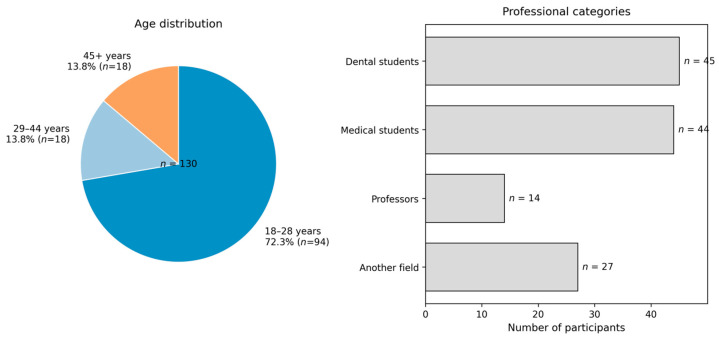
Demographic Characteristics of Study Participants.

The youngest cohort demonstrated minimal differential responses between Text 1 (faculty-authored) and Text 2 (AI-generated) across most dimensions, with the notable exception of clarity and usefulness. For Text 1, participants in the 18–28 age group evaluated clarity at 4.15, accuracy at 4.09, structure at 3.98, readability at 3.76, and usefulness at 3.95. Upon transition to Text 2, clarity increased to 4.31 (Δ = +0.15), representing the most substantial improvement observed in this cohort. Usefulness declined from 3.95 to 3.69 (Δ = −0.26), constituting the sole dimension demonstrating a decrease exceeding 0.20 points. Structure decreased minimally, accuracy increased modestly and readability decreased slightly. This pattern indicates that for Generation Z participants, the AI-generated text’s structural enhancements and clarity improvements were offset by perceived reductions in practical applicability, suggesting that younger participants may prioritize comprehensibility while simultaneously questioning the utility of reorganized content (Figure 1).

#### 3.3.2. Age Group 29–44 Years (Millennials)

The middle-aged cohort exhibited the most pronounced overall improvement from Text 1 to Text 2, demonstrating substantial gains across multiple dimensions. For Text 1, this group provided notably lower baseline ratings compared to other cohorts, with accuracy at 3.50, clarity at 3.83, structure at 3.72, usefulness at 3.61, and readability at 3.61. Upon evaluation of Text 2, accuracy increased substantially to 4.11 (Δ = +0.61), representing the single largest within-group improvement across all age cohorts and dimensions. Structure improved by 0.28 points (3.72→4.00), clarity increased by 0.28 points (3.83→4.11), and readability improved by 0.22 points (3.61→3.83). Usefulness remained essentially unchanged (3.61→3.55, Δ = −0.06). This pattern is significant: the 29–44 age group’s initially low evaluation of Text 1’s accuracy (0.55 points below the 45+ group’s evaluation) was substantially remedied by Text 2, suggesting that Millennial professionals found the faculty-authored format confusing regarding factual content but appreciated the AI model’s clarification and restructuring. The magnitude of improvement in accuracy (0.61 points) represents a fundamental shift in perceived content reliability for this cohort (Figure 1).

#### 3.3.3. Age Group 45+ Years (Generation X/Baby Boomers)

The oldest cohort demonstrated the highest absolute ratings for Text 1 across most dimensions but exhibited substantial and differential preference for Text 2 on subjective dimensions. For Text 1, participants aged 45+ evaluated accuracy at 4.39 (the highest accuracy rating across all groups and texts), clarity at 4.22, structure at 3.77, readability at 3.83, and usefulness at 3.66. Upon evaluation of Text 2, the most pronounced improvements occurred in subjective and utility-based dimensions rather than objective quality metrics. Usefulness increased dramatically from 3.66 to 4.33 (Δ = +0.67), the largest usefulness improvement across all age groups and representing a fundamental shift in perceived practical applicability. Readability increased substantially from 3.83 to 4.33 (Δ = +0.50), demonstrating that senior professionals were substantially more satisfied with the AI-generated text’s aesthetic and stylistic properties. Structure improved by 0.55 points (3.78→4.33). Accuracy decreased from 4.39 to 4.16 (Δ = −0.23), and clarity remained essentially unchanged (4.22→4.27, Δ = +0.05). This pattern reveals that while senior professionals maintained their already-elevated accuracy assessments across both texts, they derived substantially greater utility and aesthetic satisfaction from the AI-generated, restructured format, suggesting that the organizational enhancements and structured presentation resonated profoundly with this demographic (Figure 1).

### 3.4. Interferential Statistical Results

The Wilcoxon Signed-Ranks analysis examined how participants’ evaluations differed between the faculty-authored version and the AI-generated version across five pedagogical dimensions. For the structure dimension, the Z-value of −0.144 and a two-tailed significance of 0.885 indicate that the distribution of positive versus negative rank differences is essentially symmetrical (positive ranks = 47, negative ranks = 45), revealing no reliable change in perceived structural quality. Accuracy likewise showed a non-significant shift (Z = −1.232, *p* = 0.218); although more participants rated the AI text higher (positive ranks = 35) than lower (negative ranks = 24), the magnitude of this difference does not reach statistical significance. Readability follows the same pattern (Z = −0.191, *p* = 0.849), with a slight excess of positive ranks (48) over negative ranks (43) that fails to constitute a meaningful effect.

In contrast, clarity produced a statistically significant result (Z = −2.107, *p* = 0.035). The number of positive rank differences (35) markedly exceeded the negative rank differences (21), indicating that participants consistently considered the AI-generated material to be clearer than the faculty version. This finding is reinforced by the larger sum of positive ranks (1043) compared with the sum of negative ranks (553), confirming a robust improvement in perceived clarity.

Usefulness did not differ significantly between the two texts (Z = −1.124, *p* = 0.261). Positive and negative ranks were perfectly balanced (both = 47), suggesting that the AI-generated version neither enhanced nor diminished the perceived practical value of the material.

The non-parametric test demonstrates that the only dimension in which the AI-generated content achieved a clear advantage is clarity; all other evaluated aspects—structure, accuracy, readability, and usefulness—showed no statistically reliable differences between the AI and faculty versions. This pattern implies that while the AI-produced text can be perceived as more lucid, its overall instructional quality remains comparable to traditional faculty-authored material across the remaining dimensions.

Effect size analysis revealed negligible to small effects across most dimensions. The largest effect was observed for clarity (r = 0.19), indicating a small-to-moderate practical difference in favor of the second text. Sign tests confirmed the direction of differences observed in Wilcoxon analyses.

**Figure 2:**
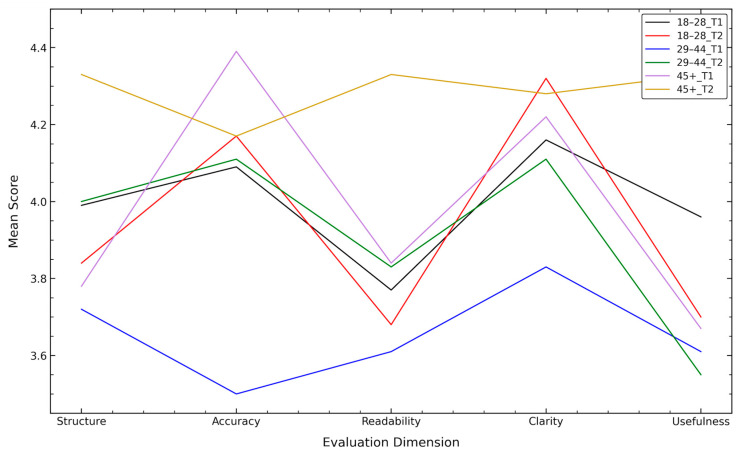
Comparison of mean scores for faculty-authored (T1) and AI-generated (T2) materials across evaluation dimensions and age groups.

**Figure 3:**
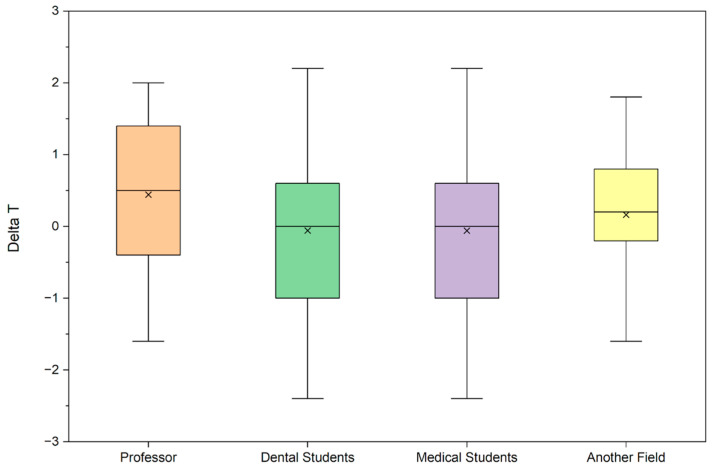
Distribution of delta scores (T2–T1) across participant groups. The box represents the interquartile range, the line inside the box indicates the median, and the “×” symbol represents the mean.

Mann–Whitney U analyses revealed no statistically significant differences in the difference score (delta_total) between the AI-generated and faculty-authored texts across participant groups. Specifically, no significant difference was observed between medical and dental students (U = 986.5, Z = −0.03, *p* = 0.992), with nearly identical mean ranks and a negligible effect size (r = 0.003). Similarly, comparisons between professors and students showed no statistically significant difference (U = 462.5, Z = −1.55, *p* = 0.122). Although professors exhibited slightly higher mean ranks, the associated effect size was small (r = 0.15) (Figure 2 and Figure 3).

A Friedman test revealed significant differences among the five evaluated dimensions of the AI-generated text (χ^2^ (4) = 43.66, *p* < 0.001). Clarity obtained the highest mean rank, followed by accuracy and structure, whereas usefulness and readability were rated lower. Kendall’s coefficient of concordance indicated a low level of agreement among participants (W = 0.084), suggesting that while differences were statistically significant, their overall magnitude was small.

Post hoc Wilcoxon signed-rank tests with Bonferroni correction demonstrated that clarity was rated significantly higher than structure in the AI-generated text (Z = −4.09, *p* < 0.001, r = 0.36).

## 4. Discussion

The comparative analysis of faculty-authored and AI-generated educational materials in dental prostheses technology revealed distinct generational patterns in how learners perceived the two versions, underscoring the importance of tailoring AI-enhanced curricula to the expectations and needs of diverse age cohorts. Among the youngest participants (Generation Z, ages 18–28), the AI-generated text was considered to be clearer than the traditional version, with a modest increase in the clarity rating that was statistically significant [45]. This improvement in readability was offset by a notable decline in perceived usefulness, suggesting that younger learners value comprehensibility but remain skeptical about the practical relevance of content that is reorganized by artificial intelligence [46]. Their assessments of accuracy, structure, and readability showed only minor fluctuations, indicating that the core scientific fidelity of the material was largely maintained across formats. This paradoxical response may reflect the digital-native orientation of Generation Z, who are accustomed to highly visual and interactive learning environments and therefore expect AI-mediated content to not only be clear but also to offer immediate applicability to clinical practice [47].

In contrast, the middle-aged cohort (Millennials, ages 29–44) exhibited the most pronounced gains in several dimensions when exposed to the AI-generated text. Accuracy improved by more than half a point, while clarity, structure, and readability each increased by roughly 0.28 points, representing the largest within-group improvements observed across all ages. These changes were accompanied by a modest, non-significant dip in usefulness, indicating that while the AI-enhanced material was perceived as more reliable and easier to follow, its practical value remained essentially unchanged for this group. The substantial elevation in accuracy ratings suggests that the restructuring and tabular presentation introduced by the language model may have clarified nuanced technical details that were less explicit in the original narrative, thereby strengthening the perceived credibility of the information among learners who are often balancing clinical responsibilities with ongoing education.

The oldest participants (Generation X and Baby Boomers, ages 45 and above) entered the study with the highest baseline scores for accuracy, clarity, and readability in the faculty-authored version, reflecting extensive professional experience and familiarity with conventional textbook formats. When evaluating the AI-generated text, this cohort reported high enhancements in subjective dimensions, with usefulness increasing by two-thirds of a point and readability rising by half a point. Structure also improved appreciably, whereas accuracy experienced a slight, non-significant decline. The pronounced boost in perceived usefulness and aesthetic satisfaction among senior professionals suggests that the organized, table-rich format produced by the language model resonated strongly with learners who value efficiency and clear visual hierarchy in dense technical material. Their willingness to accept a small reduction in accuracy highlights a pragmatic orientation toward educational resources that streamline information retrieval and support decision-making in clinical settings.

It should be noted, however, that the age-group analyses reported above are exploratory in nature and should be interpreted with caution. The unequal distribution of participants across age cohorts (with 72.3% of the sample falling within the 18–28 years group) limits the statistical comparability of between-group findings. The study was not powered a priori to detect correlations between evaluation scores and age group, and the between-group differences described above do not reach statistical significance in most dimensions. These analyses are therefore presented as preliminary, hypothesis-generating observations rather than confirmatory findings, and replication in larger, more balanced samples is required before generational conclusions can be drawn.

Statistical testing reinforced these generational trends. The Wilcoxon signed-rank analysis identified clarity as the sole dimension with a statistically significant advantage for the AI-generated text, confirming that across the entire sample participants consistently considered the AI version to be more lucid. Effect-size calculations indicated a small-to-moderate practical difference in favor of the AI version for clarity, while other dimensions showed negligible to small effects. The Friedman test further demonstrated that, within the AI-generated material, clarity achieved the highest mean rank among evaluated dimensions, followed by accuracy and structure, with usefulness and readability receiving lower rankings. Kendall’s coefficient of concordance revealed low agreement among participants, suggesting that individual preferences and expectations contributed to variability in responses [47,48,49].

The analysis of participants’ responses to the open-answer evaluative items highlights a nuanced pattern of perceived strengths and limitations of the AI-generated material, suggesting areas of pedagogical value as well as aspects requiring further refinement. Across age groups and professional categories, clarity consistently emerged as the most salient advantage of the AI-generated text. This finding indicates that the restructuring and reformulation processes employed by the language model successfully enhanced the accessibility of complex prosthodontic concepts, particularly by reducing syntactic density and improving logical flow. However, the same characteristics that contributed to increased clarity also appear to have introduced certain limitations, especially with regard to perceived practical usefulness.

Among younger participants, particularly those in the early stages of academic training, the AI-generated material was frequently described as easier to understand and more straightforward to navigate. These respondents did not uniformly associate improved clarity with increased usefulness, suggesting that simplification and reorganization alone may not fully address the need for applied, exam-oriented, or clinically anchored information. This pattern implies that while AI-generated texts can effectively support initial comprehension, they may require further adaptation to better align with performance-driven learning objectives typical of undergraduate education [48].

Participants in mid-career stages demonstrated a more balanced evaluation, recognizing both the clarity and the perceived accuracy of the AI-generated content. For this group, the strengths of the AI material appear to lie in its ability to consolidate existing knowledge and to present information in a manner conducive to rapid review and conceptual integration. At the same time, some responses suggest that excessive streamlining may risk attenuating the depth or contextual richness expected by learners who already possess foundational expertise [50].

Older participants and senior professionals tended to evaluate the AI-generated text through the lens of efficiency and readability rather than novelty or instructional innovation [51]. Their responses also underscore the importance of preserving disciplinary rigor and conventional academic framing, indicating that acceptance of AI-generated materials is contingent upon their alignment with established professional standards [52,53].

The originality of the current research derives from its controlled, blinded comparison of faculty-authored and LLM-restructured prosthodontic educational material generated from the same source chapters, with standardized formatting and removal of visual elements to ensure a strictly textual evaluation. By isolating structural reorganization as the primary variable, the design enables a focused assessment of whether AI-driven reformulation alone can influence perceived instructional quality in a technically demanding dental discipline. In addition, the integration of age-based and professional cohort stratification introduces a learner-centered analytical framework, demonstrating that the pedagogical impact of AI-generated content varies according to generational and experiential factors. This approach extends existing literature beyond general AI perception studies and provides discipline-specific empirical evidence relevant to curriculum development in dental education.

Several limitations of the present study warrant acknowledgment. First, the use of convenience sampling restricts the generalizability of findings to broader student and faculty populations. Second, the unequal group sizes—particularly the underrepresentation of faculty members (*n* = 14) relative to student groups—limit the statistical power of between-group comparisons and should be addressed in future studies through stratified or quota-based sampling designs. Third, the questionnaire instrument was developed specifically for this study rather than adapted from a previously validated tool; although internal consistency was assessed, formal construct validity and pilot testing were not conducted, which may affect the reliability of the dimensional scores. Fourth, reading time was not controlled or recorded, introducing the possibility that differential engagement with the two texts influenced evaluative responses. Finally, the study evaluated a single AI model (ChatGPT 5.2) applied to a specific textbook domain; findings may not generalize to other LLMs, prompt configurations, or dental subspecialties.

Future research should address these limitations through larger, stratified samples with balanced group representation, enabling adequately powered between-group and age-group comparisons. Longitudinal designs examining the effect of AI-generated materials on actual learning outcomes, rather than perceived quality, would substantially strengthen the evidence base. Validated instruments for the assessment of AI-generated educational content should be developed and tested across multiple dental disciplines. Additionally, the inclusion of image-rich and multimodal versions of AI-generated materials would allow for a more ecologically valid assessment of their instructional potential. Comparative studies across different LLM platforms and prompt strategies would further clarify the conditions under which AI-generated content can most effectively supplement or complement faculty-authored educational resources in dental and medical education.

## 5. Conclusions

Based on the findings of the present study, we conclude that:

1. AI-generated educational materials demonstrate pedagogical equivalence to faculty authored texts, with a distinct advantage in clarity.

Across the evaluated dimensions, the AI-generated prosthodontics material was perceived as comparable to traditional faculty-authored content in terms of accuracy, structure, readability, and usefulness, while consistently outperforming it in clarity. Large language models can effectively restructure complex disciplinary content without compromising scientific integrity, positioning clarity as their primary educational contribution.

2. Perceived benefits of AI-generated content are strongly modulated by learners’ age and professional experience.

The study reveals a clear generational gradient in how AI-restructured material is evaluated. Younger learners prioritize readability and ease of comprehension but remain cautious about practical applicability, whereas mid-career participants derive substantial benefit from improvements in perceived accuracy and organization. Senior professionals, in contrast, value efficiency, streamlined presentation, and enhanced usability, highlighting that the educational impact of AI is not uniform but context-dependent.

3. Clarity emerges as the dominant strength of AI-generated materials, while usefulness remains the most context-sensitive dimension.

Both non-parametric comparisons and within-text dimensional analyses indicate that clarity is the most salient and statistically robust advantage of AI-generated content. However, improvements in clarity do not automatically translate into higher perceived usefulness, particularly among less experienced learners. This dissociation underscores the need to distinguish between cognitive accessibility and applied relevance when evaluating AI-enhanced educational resources.

4. AI-generated texts are best positioned as complementary, rather than substitutive, educational tools.

The absence of strong group-based differences and the small-to-moderate effect sizes observed across analyses suggest that AI-generated materials neither disrupt nor diminish traditional instructional quality. Instead, they function optimally as adjunct resources that enhance comprehension, facilitate rapid review, and improve information organization, especially when integrated alongside faculty-designed curricula.

5. Effective integration of large language models in medical and dental education requires adaptive, learner-centered implementation strategies.

The divergent evaluative patterns observed across age cohorts and professional roles indicate that a one-size-fits-all approach to AI-assisted education is unlikely to be effective. Future curricular applications should combine AI-driven restructuring with explicit clinical anchoring, case-based contextualization, and supplementary materials tailored to learners’ developmental stages and professional needs, thereby maximizing educational value while preserving disciplinary rigor.

## Figures and Tables

**Table 1:** Distribution of participants across age groups and professional categories.

Category	18–28	29–44	45+	Total
Another field	19	3	5	27
Dental students	45	0	0	45
Medical students	30	12	2	44
Professors	0	3	11	14
Total	94	18	18	130

**Table 2:** Mean scores (±standard deviation) for Text 1 (faculty-authored) and Text 2 (LLM-generated) across evaluated dimensions.

Dimension	Text 1 Mean (SD)	Text 2 Mean (SD)	Δ (T2 − T1)
Clarity	4.12 (0.89)	4.28 (0.82)	+0.16
Accuracy	4.05 (0.91)	4.16 (0.88)	+0.11
Structure	3.91 (0.94)	3.97 (0.90)	+0.06
Usefulness	3.82 (1.02)	3.76 (1.08)	−0.06
Readability	3.75 (1.16)	3.83 (1.09)	+0.08

## Data Availability

The original contributions presented in the study are included in the article; further inquiries can be directed to the corresponding authors.

## Abbreviations

The following abbreviations are used in this manuscript:

AI	Artificial Intelligence
LLM	Large Language Models

## References

[1] Abd-Alrazaq A., AlSaad R., Alhuwail D., Ahmed A., Healy P.M., Latifi S., Aziz S., Damseh R., Alabed Alrazak S., Sheikh J. (2023). Large Language Models in Medical Education: Opportunities, Challenges, and Future Directions. JMIR Med. Educ..

[2] Ahsan Z. (2025). Integrating artificial intelligence into medical education: A narrative systematic review of current applications, challenges, and future directions. BMC Med. Educ..

[3] Ali K., Barhom N., Tamimi F., Duggal M. (2024). ChatGPT-A double-edged sword for healthcare education? Implications for assessments of dental students. Eur. J. Dent. Educ..

[4] Altintas L., Sahiner M. (2024). Transforming medical education: The impact of innovations in technology and medical devices. Expert Rev. Med. Devices.

[5] Amiri H., Peiravi S., Rezazadeh Shojaee S.S., Rouhparvarzamin M., Nateghi M.N., Etemadi M.H., ShojaeiBaghini M., Musaie F., Anvari M.H., Asadi Anar M. (2024). Medical, dental, and nursing students’ attitudes and knowledge towards artificial intelligence: A systematic review and meta-analysis. BMC Med. Educ..

[6] Cozmescu A.F., Cernega A., Mincă D.G., Didilescu A.C., Imre M.M., Totan A.R., Pârvu S., Pițuru S.M. (2025). Embracing Artificial Intelligence in Dental Practice: An Exploratory Study of Romanian Clinicians’ Perspectives and Experiences. Dent. J..

[7] El-Hakim M., Anthonappa R., Fawzy A. (2025). Artificial Intelligence in Dental Education: A Scoping Review of Applications, Challenges, and Gaps. Dent. J..

[8] Buduru S., Cofar F., Mesaroș A., Tăut M., Negucioiu M., Almășan O. (2024). Perceptions in Digital Smile Design: Assessing Laypeople and Dental Professionals’ Preferences Using an Artificial-Intelligence-Based Application. Dent. J..

[9] Corsello J., Nease D.B., Munie S., Bown P., Amiri F. (2022). The Importance of the Institution of a Robotic Curriculum on Resident Training and Performance. Am. Surg..

[10] Eysenbach G. (2023). The Role of ChatGPT, Generative Language Models, and Artificial Intelligence in Medical Education: A Conversation with ChatGPT and a Call for Papers. JMIR Med. Educ..

[11] Jackson P., Ponath Sukumaran G., Babu C., Tony M.C., Jack D.S., Reshma V.R., Davis D., Kurian N., John A. (2024). Artificial intelligence in medical education—Perception among medical students. BMC Med. Educ..

[12] Revilla-León M., Gómez-Polo M., Vyas S., Barmak A.B., Özcan M., Att W., Krishnamurthy V.R. (2022). Artificial intelligence applications in restorative dentistry: A systematic review. J. Prosthet. Dent..

[13] Vaswani A., Shazeer N., Parmar N., Uszkoreit J., Jones L., Gomez A., Kaiser L., Polosukhin I. (2017). Attention Is All You Need. arXiv.

[14] Minaee S., Mikolov T., Nikzad N., Chenaghlu M., Socher R., Amatriain X., Gao J. (2024). Large language models: A survey. arXiv.

[15] Mienye I.D., Jere N., Obaido G., Ogunruku O.O., Esenogho E., Modisane C. (2025). Large language models: An overview of foundational architectures, recent trends, and a new taxonomy. Discov. Appl. Sci..

[16] Xing W., Nixon N., Crossley S., Denny P., Lan A., Stamper J., Yu Z. (2025). The Use of Large Language Models in Education. Int. J. Artif. Intell. Educ..

[17] Beale R. (2025). The revolution has arrived: What the current state of large language models in education implies for the future. arXiv.

[18] Yan L., Sha L., Zhao L., Li Y., Martinez-Maldonado R., Chen G., Li X., Jin Y., Gasevic D. (2023). Practical and ethical challenges of large language models in education: A systematic scoping review. Br. J. Educ. Technol..

[19] Puleio F., Lo Giudice G., Bellocchio A.M., Boschetti C.E., Lo Giudice R. (2024). Clinical, Research, and Educational Applications of ChatGPT in Dentistry: A Narrative Review. Appl. Sci..

[20] Almalki A., Althubaitiy R.O., Alkhtani F., Anadioti E., Abozaed H.W. (2025). Assessment of ChatGPT’s Performance on the ACP 2024 National Prosthodontics Resident Exam (NPRE). Eur. J. Dent. Educ..

[21] Dashti M., Khosraviani F., Azimi T., Hefzi D., Ghasemi S., Fahimipour A., Zare N., Khurshid Z., Habib S.R. (2025). Assessing ChatGPT-4’s performance on the US prosthodontic exam: Impact of fine-tuning and contextual prompting vs. base knowledge, a cross-sectional study. BMC Med. Educ..

[22] Karan-Romero M., Salazar-Gamarra R.E., Leon-Rios X.A. (2023). Evaluation of Attitudes and Perceptions in Students about the Use of Artificial Intelligence in Dentistry. Dent. J..

[23] Shool S., Adimi S., Saboori Amleshi R., Bitaraf E., Golpira R., Tara M. (2025). A systematic review of large language model (LLM) evaluations in clinical medicine. BMC Med. Inform. Decis. Mak..

[24] Cascella M., Montomoli J., Bellini V., Bignami E. (2023). Evaluating the Feasibility of ChatGPT in Healthcare: An Analysis of Multiple Clinical and Research Scenarios. J. Med. Syst..

[25] Simoni J., Urtubia-Fernandez J., Mengual E., Simoni D.A., Royo M., Egaña-Yin D., Hertog O.L.A., López-Ortiz L., Muñoz-Tomás A., Santiago-Martínez P. (2025). Artificial intelligence in undergraduate medical education: An updated scoping review. BMC Med. Educ..

[26] Boscardin C.K., Gin B., Golde P.B., Hauer K.E. (2024). ChatGPT and Generative Artificial Intelligence for Medical Education: Potential Impact and Opportunity. Acad. Med..

[27] Salvagno M., Taccone F.S., Gerli A.G. (2023). Can artificial intelligence help for scientific writing?. Crit. Care.

[28] Karaca O., Çalışkan S.A., Demir K. (2021). Medical artificial intelligence readiness scale for medical students (MAIRS-MS)—Development, validity and reliability study. BMC Med. Educ..

[29] Lee H. (2024). The rise of ChatGPT: Exploring its potential in medical education. Anat. Sci. Educ..

[30] Meşe İ., Altıntaş Taşlıçay C., Kuzan B.N., Kuzan T.Y., Sivrioğlu A.K. (2024). Educating the next generation of radiologists: A comparative report of ChatGPT and e-learning resources. Diagn. Interv. Radiol..

[31] Sivaramakrishnan G., Almuqahwi M., Ansari S., Lubbad M., Alagamawy E., Sridharan K. (2025). Assessing the power of AI: A comparative evaluation of large language models in generating patient education materials in dentistry. BDJ Open.

[32] Yüzbaşıoğlu E. (2021). Attitudes and perceptions of dental students towards artificial intelligence. J. Dent. Educ..

[33] Tangadulrat P., Sono S., Tangtrakulwanich B. (2023). Using ChatGPT for Clinical Practice and Medical Education: Cross-Sectional Survey of Medical Students’ and Physicians’ Perceptions. JMIR Med. Educ..

[34] Thomae A.V., Witt C.M., Barth J. (2024). Integration of ChatGPT Into a Course for Medical Students: Explorative Study on Teaching Scenarios, Students’ Perception, and Applications. JMIR Med. Educ..

[35] Triola M.M., Rodman A. (2025). Integrating Generative Artificial Intelligence Into Medical Education: Curriculum, Policy, and Governance Strategies. Acad. Med..

[36] Weidener L., Fischer M. (2024). Artificial Intelligence in Medicine: Cross-Sectional Study Among Medical Students on Application, Education, and Ethical Aspects. JMIR Med. Educ..

[37] Ciocan L.T. (2025). Removable and Mobile Dental Prostheses Technology.

[38] Cecconi C., Adams R., Cardone A., Declaye J., Silva M., Vanlerberghe T., Guldemond N., Devisch I., van Vugt J. (2025). Generational differences in healthcare: The role of technology in the path forward. Front. Public Health.

[39] Traboulsy S., Demian J., Tamim H., Hadid D., Hitti E. (2025). Generational differences in career paths and effort allocation of graduates of a top-research medical school, Lebanon. BMC Med. Educ..

[40] Papp-Zipernovszky O., Horváth M.D., Schulz P.J., Csabai M. (2021). Generation Gaps in Digital Health Literacy and Their Impact on Health Information Seeking Behavior and Health Empowerment in Hungary. Front. Public Health.

[41] Saxena S., Tsobgnie M.N., Südy R., Gisselbaek M., Lechien J.R., Carella M., Ingrassia P.L., Dieckmann P., Berger-Estilita J. (2026). Generation Z versus generative artificial intelligence: A cross-sectional study assessing medical students’ confidence and over-reliance on artificial intelligence in perioperative clinical scenarios. Eur. J. Anaesthesiol..

[42] Lalani K., Butler-Henderson K., Marc D.T., Fenton S.H. (2025). Generational Health Professional Perceptions About Artificial Intelligence Impact on Clinical Work. Stud. Health Technol. Inform..

[43] Gallagher V.T., Reilly S.E., Martin D., Manning C., Shaffer K.M. (2024). Examining Differences in Health-Related Technology Use between Millennial and Older Generations of Caregivers. Nurs. Rep..

[44] Granjeiro J.M., Cury A., Cury J.A., Bueno M., Sousa-Neto M.D., Estrela C. (2025). The Future of Scientific Writing: AI Tools, Benefits, and Ethical Implications. Braz. Dent. J..

[45] McCoy L., Ganesan N., Rajagopalan V., McKell D., Niño D.F., Swaim M.C. (2025). A Training Needs Analysis for AI and Generative AI in Medical Education: Perspectives of Faculty and Students. J. Med. Educ. Curric. Dev..

[46] Kung T.H., Cheatham M., Medenilla A., Sillos C., De Leon L., Elepaño C., Madriaga M., Aggabao R., Diaz-Candido G., Maningo J. (2023). Performance of ChatGPT on USMLE: Potential for AI-assisted medical education using large language models. PLoS Digit. Health.

[47] Miranda S. (2025). Artificial Intelligence in Education: An Exploratory Survey to Gather the Perceptions of Teachers, Students, and Educators Around the University of Salerno. Educ. Sci..

[48] Adel A., Ahsan A., Davison C. (2024). ChatGPT Promises and Challenges in Education: Computational and Ethical Perspectives. Educ. Sci..

[49] Garzón J., Patiño E., Marulanda C. (2025). Systematic Review of Artificial Intelligence in Education: Trends, Benefits, and Challenges. Multimodal Technol. Interact..

[50] Mutanga M.B., Jugoo V., Adefemi K.O. (2024). Lecturers’ Perceptions on the Integration of Artificial Intelligence Tools into Teaching Practice. Trends High. Educ..

[51] Nasra M., Jaffri R., Pavlin-Premrl D., Kok H.K., Khabaza A., Barras C., Slater L.A., Yazdabadi A., Moore J., Russell J. (2025). Can artificial intelligence improve patient educational material readability? A systematic review and narrative synthesis. Intern. Med. J..

[52] Keating M., Bollard S.M., Potter S. (2024). Assessing the Quality, Readability, and Acceptability of AI-Generated Information in Plastic and Aesthetic Surgery. Cureus.

[53] Ding S.R., Ahmed M., Malik T., Somagani R., Vohra F.F. (2025). Readability Comparison of AI-Generated Versus UpToDate Educational Content on Stroke Management: A Cross-Sectional Study. Cureus.

